# A Review on Comfort of Pedestrian Bridges Under Human-Induced Vibrations and Tuned Mass Damper Control Technologies

**DOI:** 10.3390/ma18163903

**Published:** 2025-08-21

**Authors:** Shoukun Zhang, Baijin Wu, Yong Tang, Han Zhang, Zheng Xu, Guoqiang Li, Shuang Lu

**Affiliations:** 1 School of Civil Engineering, Harbin Institute of Technology, Harbin 150006, China; zhangshoukun2005@163.com (S.Z.); 15951599925@163.com (B.W.); lus@hit.edu.cn (S.L.); 2 NO. Three Engineering Co., Ltd. of CCCC First Highway Engineering Co., Ltd., Beijing 101102, China; maimaituq@163.com (Y.T.); lv_maorong@163.com (H.Z.); 3 College of Ecology and Environment, Xinjiang University, Urumqi 830046, China; 4 School of Physics and Materials Science, Changji University, Changji 831100, China

**Keywords:** pedestrian bridge, comfort, anthropogenic vibration, pedestrian load modeling, normative documents, damping design, tuned mass damper

## Abstract

With the development of urban infrastructure construction, while pedestrian bridges meet traffic functions the issue of their comfort has become a core consideration in structural design. This is because the long-span lightweight structures, with their large flexibility and low fundamental frequencies, are also vulnerable to human-induced vibrations. Pedestrian load modellings include the deterministic time-domain model, which is widely adopted in codes due to its simplicity, the random model that takes into account individual variability, and the frequency-domain model. The deterministic time-domain model has abundant parameter determination results and has become relatively mature, while the latter two, although more rigorous, have relatively lagging development. Numerous studies have shown that acceleration limits are the main indicators for comfort assessment. Vertical vibrations are controlled by amplitude constraints, while for the lateral vibrations the “lateral lock-in” that can cause dynamic instability needs to be evaluated with particular emphasis. When comfort exceeds an acceptable degree, a prevalent countermeasure is to attach a Tuned Mass Damper (TMD) or Multiple Tuned Mass Damper (MTMD) system to the structure—the latter demonstrates stronger robustness when dealing with random pedestrian loads.

## 1. Introduction

With the development of urban infrastructure construction, pedestrian bridges are becoming more and more important in urban transportation networks, and their functions are gradually integrating landscape attributes rather than pure transportation needs. Driven by engineering technology and architectural aesthetics, the wide application of lightweight and high-strength materials has made footbridges develop towards large-span and shaped structure [1,2,3,4]. At the same time, this also brings the characteristics of large structural flexibility and low fundamental frequency—when the pedestrian’s step frequency is close to the bridge’s intrinsic frequency it is easy to trigger resonance, generating excessive vertical or transverse vibration, which not only affects the comfort and safety of pedestrians, but may even lead to structural dynamic instability and may also cause the bridge to be damaged by the vibration [5,6]. Examples such as the incident of the Millennium Bridge in London, which was temporarily closed due to significant lateral vibrations triggered by the crowd load, have highlighted the importance of human-caused vibrations and triggered a large number of related studies.

Currently, the research on pedestrian bridge human-induced vibrations mainly focuses on three aspects:

1. Pedestrian load modeling. Single-person walking load has significant randomness in individual weight, step frequency, gait, and other differences, and there are changes in pedestrian density and other environmental factors. Crowd loading is a more complex nonlinear role and needs to simplify the assumptions in modeling.
2. Vibration response evaluation system. Existing research has been initially established to acceleration-limit-value-oriented comfort standards, avoiding sensitive frequency intervals and limiting the dynamic response control vibration, but lateral vibration comfort research is less and more concentrated in high-rise buildings. Lateral vibration also needs to be vigilant about the “load–response” nonlinear feedback triggered by the dynamics of the instability problem.
3. Optimization of vibration control technology. Adjustment of structural stiffness is limited due to cost and aesthetic constraints, while a tuned mass damper (TMD) has become a mainstream in engineering due to its simple structure, high damping efficiency, and easy installation. However, a single TMD has insufficient adaptability to face broadband loading, and a multi-tuned mass damper (MTMD), although theoretically more applicable to broadband excitation, needs to balance the computational complexity of the optimization of the parameters with the feasibility of the engineering.

Several national and regional design codes have proposed vibration comfort calculation methods and standards. The European EN 1990 [7] points out that when the pedestrian step frequency is close to the intrinsic frequency of the bridge it is necessary to carry out vibration response analysis, and it specifies the maximum limit of vertical and lateral acceleration. The French Sétra for the assessment of the vibrational behavior of footbridges under pedestrian loading [8] (referred to as the “Sétra”) puts forward stricter peak acceleration assessment criteria and sensitive frequency interval provisions. The United States and Canada have proposed more stringent peak acceleration assessment criteria and sensitive frequency interval provisions. However, there are still differences in the evaluation methods of the national standards, for example, the European and French norms focus on vertical and lateral acceleration control while China’s “Technical specification of urban pedestrian overcrossing and underpass” [9] (CJJ69-95) is mainly based on the control of vertical frequency to avoid resonance.

In this study, previous relevant studies and design codes from various countries and regions were collected. Various pedestrian load models were reviewed and their characteristics were summarized. This process serves as the prerequisite and foundation for analyzing human-induced vibration. Regarding serviceability issues, numerous scholars and engineers recognize the relationship these issues have with both structural dynamic characteristics and dynamic responses. Extensive and instructive research has been carried out, with findings incorporated into design codes. Therefore, it is necessary to review these research outcomes in conjunction with typical design codes to explore serviceability criteria. Furthermore, the representative theoretical research on the design methods of the TMD and MTMD systems was introduced, which are critical devices for ensuring serviceability. The findings reviewed and highlighted are more likely to be effective in engineering practice, aiming to provide references for engineering design.

## 2. Pedestrian Load

Pedestrian load has clear characteristics:

1. Periodicity: Although there are slight differences in the length and frequency of a single step of pedestrians, the inherent law of human walking determines that the walking loads are significantly periodic, and the time series of their forces show regular changes.
2. Narrow-band randomness: The weight, step frequency, step length, and other parameters of different pedestrians differ, but the fluctuation range is small, resulting in a random distribution of the load parameters in a narrow interval.
3. Human–bridge interaction: Pedestrians are not only the excitation source of bridge vibration but are also affected by the dynamic response of the bridge. This makes them adjust their walking posture, which in turn changes the load acting on the bridge, forming a dynamic feedback of “load–response”; at the same time, the real-time changes in the number, density, and location of pedestrians on the bridge surface make the dynamic load present a dynamic characteristic, so the crowd is very important for the accurate analysis of bridge dynamics. Meanwhile, the real-time changes in the number, density, and location of pedestrians on the bridge make the dynamic load show dynamic characteristics, so crowd load study is crucial to accurately analyze the dynamic response of bridge.

### 2.1. Single Pedestrian Load

**Figure 1:**
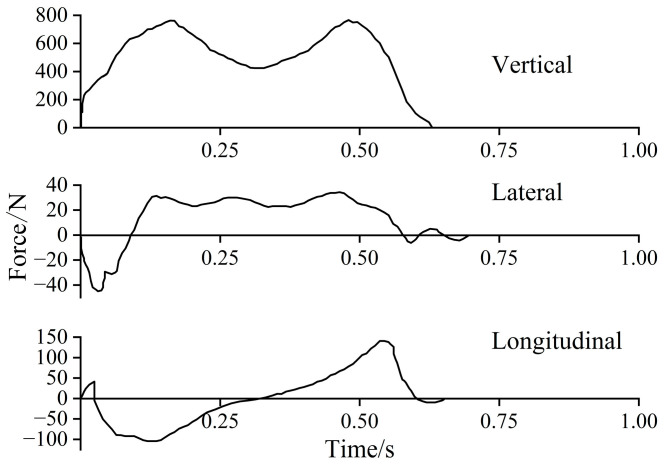
Time–history curves of single-step force [10].

Walking is the process of supporting the center of gravity of the human body by alternating between the two legs. When walking, the left and right feet land alternately, driving the center of gravity of the human body to move up and down and move left and right in an approximate cyclical change. Vertically, each step constitutes a complete cycle; horizontally and laterally, the left and right feet need to step out to complete a cycle. This cyclic motion results in dynamic time-varying walking forces in three directions, including vertical, horizontal lateral, and horizontal longitudinal forces, for a single-person walking. Andriacchi et al. [10] conducted real measurements of walking loads using force plates to obtain typical single-step time-varying curves, as shown in Figure 1.

Continuous walking loads can be synthesized based on this result, ignoring subtle differences and taking into account the overlap time of the feet. Since then, many researchers [11,12,13,14] have used force plates to carry out walking load measurement studies, and although the single-step time–distance curves obtained are somewhat different due to the number of samples and different testing conditions, the overall pattern is basically the same.

To accurately assess the dynamic performance and vibration control effect of the footbridge during service, it is necessary to establish a reasonable single-person walking load model based on a large number of single-step load tests and then further construct a crowd load model. In the construction of the load model, two requirements should be taken into account: from the purpose of use, the model should be as close as possible to the real situation to correctly reflect the dynamic response of the footbridge; from the consideration of the use of the performance, the model needs to be reasonably simplified to facilitate the application of engineering design. Based on the consideration of the balance of these two aspects, the established models for single pedestrians can be divided into two categories: the time-domain model and the frequency-domain model. Among them, the time-domain model can be further divided into a deterministic model and stochastic model according to the randomness of the pedestrian walking process.

#### 2.1.1. Time-Domain Model

The deterministic time-domain model treats the walking load of a single person as a continuous and stable periodic load. It does not consider the narrow-band randomness in the walking process, it ignores the difference between the left and right foot walking loads, and the step frequency is also kept constant.

The model expresses the vertical force *F_pv_*(*t*) and the horizontal force *F_ph_*(*t*) generated during the walking process as a Fourier triangular magnitude consisting of multi-order harmonics, which is presented as follows [15]:

(1) $$F_{pv}\left(t\right)=G\left[1+\sum_{i=1}^{n}\alpha_{vi}\sin\left(2\pi if_{pv}t-\varphi_{vi}\right)\right],$$

(2) $$F_{ph}\left(t\right)=G\sum_{i=1}^{n}\alpha_{hi}\sin\left(2\pi if_{ph}t-\varphi_{hi}\right),$$

where *G* is the pedestrian gravity; *i* is the number of harmonics; *n* is the total number of harmonics contributing to the load, the value of which is related to the required degree of approximation, and *n* = 1 is sufficient in general engineering practice; *f_pv_* and *f_ph_* are the step frequencies in the vertical and horizontal directions, respectively, and since the left and right feet on the horizontal transverse direction each take a step to complete a cycle, the step frequencies in the vertical direction and the horizontal longitudinal direction are twice as many as those in the horizontal transverse direction; *φ_vi_* and *φ_hi_* are the phase angles of the *i*th harmonic; *α_vi_* and *α_hi_* are the dynamic load factor (DLF) at the *i*th harmonic in the vertical and horizontal directions, respectively, and the value of the DLF is the basis of the model.

**Figure 2:**
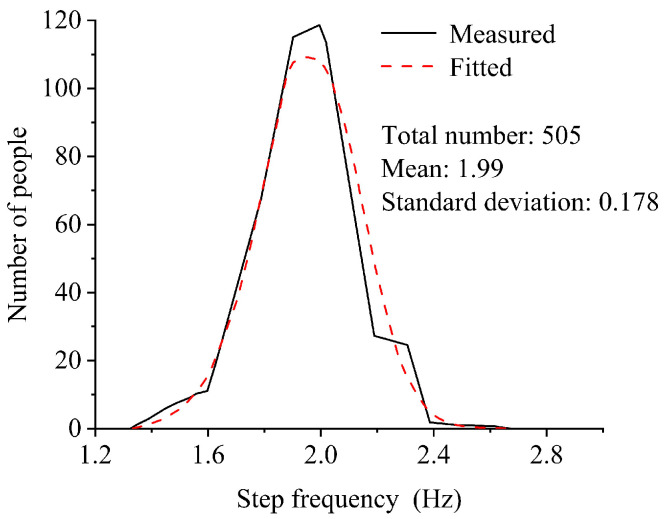
Typical statistics of pedestrian step frequency [17].

Regarding the parameters in the load model, many studies showed that the pedestrian gravity *G* obeys a normal distribution, with only a small difference in the results of statistics for people in different regions [16]. The probability description of the step frequency *f_p_* was first given by Matsumoto et al. [17], whose experimental results concluded that *f_p_* also obeys the normal distribution, with a mathematical expectation of 1.99 Hz and a standard deviation of 0.178 Hz, as shown in Figure 2. Related studies since then have also confirmed that *f_p_* obeys the result of the normal distribution [18,19].

The dynamic load factor (DLF) is a key factor in determining the walking load, which directly affects the performance of the established walking load model. Due to the different test methods, test instruments, and test objects adopted by different researchers and the differences in the walking characteristics of people and the resulting load characteristics, which lead to the differences in the values of the proposed DLF, Blanchard et al. [20] first proposed a simple resonance-based walking force model with a dynamic load factor DLF of 0.257, which is used for pedestrian bridges with a fundamental frequency of less than 4 Hz. Since the fundamental frequency above 4 Hz is difficult to be excited by the first-order component, the effect of the second-order component needs to be taken into account and some discounting should be performed, but this value is significantly smaller than the value of the DLF suggested by later researchers. Bachmann and Ammann [21] gave the first five orders of the DLF for vertical, horizontal transverse, and horizontal longitudinal directions, and concluded that the pedestrian’s stride frequency is the main factor influencing the DLF, which was also confirmed by the later studies. Rainer et al. [22] conducted a systematic study on the change in vertical force with stride frequency and obtained the relationship between each order of the DLF and stride frequency, but his study only included three testers and the results were not reliable enough. Allen et al. [23] gave a more conservative value of the DLF for a general stride frequency based on Rainer’s study. The study by Kerr et al. [18] is the most comprehensive at present; they analyzed thousands of walking force data with step frequency between 1 and 3 Hz, and the results revealed that the first-order DLF is obviously related to the pedestrian step frequency, but the second, third, and fourth values show great dispersion and so they are taken to be the statistical averages. Young [24] combined the results of the research of other scholars and proposed that the first four orders of DLF values are inversely related with the step frequency function relationship between the first four DLF values and step frequency with 75% assurance rate, and this result was also adopted by Arup’s engineering designers. Table 1 summarizes the DLF values proposed by some scholars.

Schweizer et al. [27] proposed a fuzzy mathematical theory-based approach to pedestrian load modeling that addresses the problem of multi-model parameter characterization by constructing a fuzzy Fourier series (FFS) framework to interpret Fourier series from six different authors, such as Young, Bachmann, and Kerr et al. [18,21,24], in a single model. The method addresses the variability of the DLF and phase angle in the six representative studies by using fuzzy triangular numbers/trapezoidal intervals for a unified characterization of the range of parameter values, using fuzzy probabilistic stochastic (fp-r) variables to characterize the cognitive uncertainty of the step frequency and realizing the coupled multi-level uncertainty analysis using a hybrid Monte Carlo-agent modeling algorithm.

Schweizer et al. proposed that the first three dynamic loading factors, *α*_v1_, *α*_v2_, and *α*_v3_, have functional dependence on the step frequency *f_p_* and hence are defined as fuzzy functions according to Equations *α*_v4_ and *α*_v5_ have constant bounds and hence are modeled by fuzzy numbers. See Equations (3)–(6).

(3) $$\begin{array}{l} \alpha_{v1,min}=\left\{\begin{array}{cc} 0.324f_{p}-0.3224, & f_{p}<2.22\;\mathrm{Hz}\\ 0.4, & f_{p}\geq 2.22\;\mathrm{Hz} \end{array}\right.\\ \alpha_{v1,mean}=\left\{\begin{array}{cc} 0.37(f_{p}-0.95), & f_{p}<2.3\;\mathrm{Hz}\\ 0.5, & f_{p}\geq 2.3\;\mathrm{Hz} \end{array}\right.\\ \alpha_{v1,max}=\left\{\begin{array}{cc} 0.4, & f_{p}<2.0\;\mathrm{Hz}\\ 0.37f_{p}-0.34, & f_{p}\geq 2.0\;\mathrm{Hz} \end{array}\right. \end{array}$$

(4) $$\begin{array}{l} \alpha_{v2,min}=0.05\\ \alpha_{v2,mean}=0.1\\ \alpha_{v2,max}=0.36f_{p}-0.23 \end{array}$$

(5) $$\begin{array}{l} \alpha_{v3,min}=0.025\\ \alpha_{v3,mean}=0.06\\ \alpha_{v3,max}=\left\{\begin{array}{cc} 0.1, & f_{p}<1.6\;\mathrm{Hz}\\ 0.22f_{p}-0.26, & f_{p}\geq 2.0\;\mathrm{Hz} \end{array}\right. \end{array}$$

(6) $$\tilde{\alpha_{v4}}=\langle 0\;0.05\;0.07\rangle ,\;\tilde{\alpha_{v5}}=\langle 0\;0.03\;0.06\rangle$$

Besides the narrow-band randomness, stemming from differences in various individuals (inter-subject variability), each step of a single person also has diminutive distinctions (intra-subject variability) [28]. This stochasticity leads to significant shortcomings of the ideal cyclic loading model in structural resonance analysis, especially for higher-order resonance frequencies, which can lead to design conservatism problems [29]. In terms of spectral characteristics, the amplitude spectrum of the ideal cyclic walking excitation has discrete peaks only at the step frequency and its octave, while the spectrum of the actual walking load forms a main peak near the step frequency. However, the energy spreads out to the neighboring frequencies, and this frequency dispersion effect is more prominent in the high-frequency component. This difference makes it difficult for the DLF based on the ideal cycle assumption to accurately characterize the dynamic properties of real walking loads, and exposes the shortcomings of the deterministic time-domain model in describing the stochasticity of loads.

Based on the above understanding, Živanović et al. [29] proposed the concepts of main harmonic DLF and subharmonic DLF and established a time-domain stochastic model for pedestrian loads based on considerations of group variability and individual variability. In the form of the deterministic model as a carrier, the probabilistic model is dominant in the value of walking force parameters—each parameter of walking load is regarded as a random variable and the model is closer to the actual walking force load than the deterministic time-domain model.

Živanović et al. considered the first five orders of the main harmonics and subharmonics and considered the main harmonic DLF as a normal variable. The results of Kerr et al. are used directly for the mean values of the first two orders of the main harmonic DLF; based on the statistics of the experimental data the third to fifth orders of the main harmonic DLF are determined using the method of Kerr et al., in which the sample mean is multiplied by a correction coefficient obeying a normal distribution. Then, the least squares method is used to fit the linear relationship between the first-order main harmonic DLF and the first five-order subharmonic DLFs. The correlation results are listed in Table 2, and the mean value of DLF *μ*_DLF1_ for the first-order main harmonic is:

(7) $$\mu_{\mathrm{DLF}1}=-0.2649f_{p}^{3}+1.3206f_{p}^{2}-1.757f_{p}+0.7613.$$

The normalized dynamic load factors $\bar{{\mathrm{DLF}}_{i}}\left({\bar{f}}_{j}\right)$ and ${\bar{{\mathrm{DLF}}_{i}}}^{S}\left({\bar{f}}_{j}^{S}\right)$ for the *ith* order main harmonics and subharmonics are:

(8) $$\bar{{\mathrm{DLF}}_{i}}\left({\bar{f}}_{j}\right)=\sum_{k=1}^{3}a_{i,k}e^{-\left(\frac{{\bar{f}}_{j}-b_{i,k}}{c_{i,k}}\right)},$$

(9) $${\bar{{\mathrm{DLF}}_{i}}}^{S}\left({\bar{f}}_{j}^{S}\right)=\sum_{k=1}^{2}a_{i,k}^{S}e^{-\left(\frac{{\bar{f}}_{j}^{S}-b_{i,k}^{S}}{c_{i,k}^{S}}\right)},$$

where ${\bar{f}}_{j}$ and ${\bar{f}}_{j}^{S}$ are the frequency-to-step-frequency ratios of the main and second harmonics; $a_{i,k}$, $b_{i,k}$, and $c_{i,k}$ are the fitting parameters of the main harmonics; and $a_{i,k}^{S}$, $b_{i,k}^{S}$, and $c_{i,k}^{S}$, are the fitting parameters of the second harmonics. In addition, Živanović et al. proposed that the phase at [−*π*, *π*] obeys a uniform distribution. After determining the amplitude–frequency and phase–frequency relationships, the time-domain expression of the walking load can be constructed, and the walking load components of the *i*th order main harmonics and subharmonics $F_{i}\left(t\right)$ and $F_{i}^{S}\left(t\right)$ are as follows:

(10) $$F_{i}\left(t\right)=G\cdot {\mathrm{DLF}}_{i}\cdot \sum_{\bar{f}=i-0.25}^{i+0.25}\bar{{\mathrm{DLF}}_{i}}\left(\bar{f_{j}}\right)\cos\left[2\pi {\bar{f}}_{j}f_{p}t+\theta \left({\bar{f}}_{j}\right)\right],$$

(11) $$F_{i}^{S}\left(t\right)=G\cdot {\mathrm{DLF}}_{i}^{S}\cdot \sum_{\bar{f}=i-0.25}^{i+0.25}{\bar{\mathrm{DLF}}}_{j}^{S}\left({\bar{f}}_{j}^{S}\right)\cos\left[2\pi {\bar{f}}_{j}^{S}f_{p}t+\theta \left({\bar{f}}_{j}^{S}\right)\right],$$

where *G* is the pedestrian’s gravity; *f_p_* is the pedestrian’s step frequency; and ${\mathrm{DLF}}_{i}$ and ${\mathrm{DLF}}_{i}^{S}$ are the dynamic loading factors of the *i*th order main and second harmonics, respectively. Then, the pedestrian load under the joint action of the first *n*th order main and second harmonics is:

(12) $$F\left(t\right)=\sum_{i=1}^{n}F_{i}\left(t\right)+\sum_{i=1}^{n}F_{i}^{S}\left(t\right).$$

#### 2.1.2. Frequency-Domain Model

The walking load frequency-domain model first originated from Ohlsson’s research [30], who superimposed a continuous walking force time course by using the method of period extension based on the measured single-step load, and obtained the self-power spectrum of the walking load through the spectral transformation of the time course curve. However, Ohlsson only explored the walking load in the high-frequency range above 6 Hz. Since then, Eriksson [31] added to this foundation the power spectrum of walking loads below 6 Hz. Unfortunately, the disadvantage of the above frequency-domain models is that they cannot take into account the effect of the individual variability of pedestrians. Brownjohn [32] remedied this by taking real measurements of the vertical walking forces of three test subjects and proposed the following frequency-domain model of vertical walking loads that takes into account the individual variability:

(13) $$S_{F,n}\left(f\right)={\left[WG_{n}\left(\bar{f}\right)\right]}^{2}\cdot {\left[G_{n}^{’}\left(f/\bar{f}\right)\right]}^{2}/2d\bar{f},$$

(14) $$G_{n}^{’}\left(f/\bar{f}\right)=A+Be^{-\frac{{\left|f/\bar{f}-1\right|}^{C}}{D}},$$

where *S_F_*_,*n*_ is the *nth* order harmonic of the vertical walking load; $\bar{f}$ is the average pedestrian step frequency; *G_n_*′ is the normalized Fourier amplitude fitted according to the DLF values *G_n_*; and A, B, C, and D are its fitting coefficients. Since then, Ricciardelli modeled pedestrian lateral walking loads based on measured walking load data [33], producing the following:

(15) $$\frac{S_{{\left(F_{L}\right)}_{k}}\left(f\right)f}{{\left({\tilde{F}}_{Li}^{2}\right)}_{k}}=\frac{2A_{i}}{\sqrt{2\pi }B_{i}}e^{-2{\left(\frac{f/i\bar{f}-1}{B_{i}}\right)}^{2}},$$

where $S_{{\left(F_{L}\right)}_{k}}$ is the *i*th harmonic of the lateral walking load; ${\tilde{F}}_{Li}^{2}$ is the area of the *i*th harmonic power spectral density, i.e., the harmonic energy; ${\left(.\right)}_{k}$ denotes the taking of the corresponding eigenvalues; $A_{i}$ and $B_{i}$ are the fitting parameters.

Since the narrow-band stochasticity of single-person walking dynamic forces is not fully recognized and there is significant individual variability in human–bridge interactions, pedestrian loads are difficult to be described by accurate mathematical models and current dynamic walking force models are constructed based on specific assumptions. From the characteristics of the three mainstream models, the deterministic time-domain model ignores the stochasticity of pedestrian loads and has the significant advantages of concise form and engineering applicability, the core of which lies in the determination of the DLF. However, from the research results in Table 1, it can be seen that the higher-order DLF is greatly affected by randomness, which makes the model limited in describing the complex vibration response. The walking force stochastic time-domain model takes into account the nonperiodicity of walking loads and individual differences, which is significantly better than the deterministic model in terms of model reasonableness. However, the theoretical research in this field lags behind, and only the model proposed by Živanović et al. was presented as a representative result, which limits its wide application. The frequency-domain model is an ideal way to describe the randomness of walking loads as it rigorously considers the randomness of walking loads from the theoretical level. However, due to the lag in the study of the spatial correlation of pedestrian loads the model can only be applied to the vibration analysis of pedestrian bridges under extreme working conditions, and the development of its engineering practicability is restricted.

### 2.2. Crowd Load

The core of crowd load modeling lies in the mathematical portrayal of the interactions between crowds and the spontaneous synchronization and coordination mechanism of the pedestrian bridge system, which makes the modeling process more complicated than that of single-person loads. Current studies usually classify crowd loading into the following categories: small group walking, low-density free walking, high-density crowd mobility, and the “locked” state of lateral dynamic instability. Of these, the first three can be used to calculate the maximum acceleration of a footbridge when it vibrates under un-stabilized conditions, while the last type of model can be used to determine the critical number of people that trigger dynamic instability.

Walking in a group means that about ten pedestrians pass the footbridge together in the same direction and at approximately the same speed. At this time, each of their step frequencies are very close to each other (many people will enter the synchronized state), and so the first-order harmonic component of the vertical dynamic load is easy to synchronize and the possibility of synchronization of the higher-order harmonics is small and can be ignored. At this time, the group’s footstep dynamic load effect *F_p_* can be calculated as *n* times (the number of people in the group) the first-order harmonic component:

(16) $$F_{p}=n\cdot G\cdot \alpha_{1}\cdot \sin\left(2\pi f_{p}t\right).$$

The transverse component of the human action load can also be calculated using this model.

When the density of pedestrians on the bridge is lower than 0.5 p/m^2^, pedestrians can walk freely on the bridge according to their own wishes and so walking trajectory, step length, step frequency, and walking habits are basically not affected by other people. Additionally, the bridge will not produce excessive vibrations which lead to pedestrians adjusting their walking strategy and so there is no need to consider the problem of human–bridge coupling. At this point, the problem can be simplified: *n* different pedestrians generated by the walking load equivalent to *N_p_* of the pedestrian walking synchronization generated by the walking load, i.e., *n* equals the amplitude but randomly distributed phase of the dynamic load and so the value of a single load is equivalent to $\sqrt{n}$ times, i.e., *N_p_* = $\sqrt{n}$ [34]. Bachmann and Ammann [20] argued that this equivalent number of people calculation formula is only applicable to bridges with a natural frequency in the range of 1.8–2.2 Hz, where the pedestrian step frequency is mainly distributed. For bridges with a natural frequency in the ranges of 1.6–1.8 Hz and 2.2–2.4 Hz, however, a reduction should be made through linear interpolation between 2 and $\sqrt{n}$. (*n* > 4). In 2006, the French Ministry of Transport’s technical department published Sétra [7], which used random probability distribution simulation methods combined with a large number of numerical simulation results to provide an equivalent crowd formula for considering the damping ratio *ξ* when the crowd density is less than 1 p/m^2^: *N_p_* = 10.8$\sqrt{n\cdot \xi }$.

When the density of pedestrians on the bridge exceeds 0.5 p/m^2^, the dynamic interaction between pedestrians significantly affects the walking parameters. Wei et al. [35] found through the simulation of the social force model that at a density of 0.8 p/m^2^ a pedestrian’s walking speed decreases and the global average stride frequency decreases to 1.81 Hz, and that this stride frequency concentration effect may trigger the synchronization phenomenon in advance. Additionally, when the bridge pedestrian density exceeds 1 p/m^2^, pedestrians are very close to each other and influence each other during walking, and each of them cannot decide the stride length and stride frequency, etc., completely autonomously. A study of Sétra considers that when a high density of people walk on a bridge deck the probability of synchronization of stride frequency is very high, and it adopts the same random probability distribution simulation method as that of the equivalent number of a low density of people. Furthermore, the equivalent number of a high density of people is 1.85 times of that of the literature [34], i.e., *N*_p_ = 1.85$\sqrt{n}$. The formula for the equivalent number of people given by Sétra is also later adopted by other related specifications.

### 2.3. Provisions on Pedestrian Load in Design Codes

Combined with the above research, the relevant codes in many countries have made provisions for pedestrian loads, and Table 3 lists the models of pedestrian loads given in some of the codes.

The British BS 5400 [36] only considers the first-order harmonics, and the pedestrian step frequency is taken as the vertical fundamental frequency of the structure *f*. This specification calculates the dynamic response of the pedestrian dynamic load moving from one end of the bridge to the other with the speed *V*_t_, and the moving speed of a single pedestrian dynamic load on the bridge is taken as *V*_t_ = 0.9 *f*, which means that the pedestrian step length is assumed to be 0.9 m. The pedestrian load selected by the Swedish BRO 2004 [37] is a fixed sinusoidal load, and the load model is based on the effect of crowd density. The effect of crowd density is considered. The load model takes into account the effect of crowd density, where *B* and *L* are the bridge width and span length, respectively, 0.1 is the crowd density, and *f_p_* is the pedestrian step frequency. Since the load model is fixed-point loading, which amplifies the structural dynamic response, 0.1 is still taken when the crowd density on the bridge is large. ISO 10137 [38] specified that both vertical and lateral single-person loads are in the form of Fourier series. When calculating the crowd load, the single-person load is multiplied by the number of persons *n* and then applied to the bridge in the form of a uniform load or line load. Considering the variability of the footsteps of *n* people on the bridge the dynamic response of the structure will be reduced compared to a fully coordinated crowd, so it should be discounted by multiplying it by a coordination factor; *C*(*n*) is taken as 1 only when the pedestrians are fully synchronized. In 2006, the technical department of transport road and bridge engineering and road safety under the French Ministry of Transport issued a design guideline for pedestrian bridges [8] for evaluating the vibration characteristics of pedestrian bridges under pedestrian loads, specifying that a single-person walking on a bridge is not required to initiate the vibration. It specifies a single-person-walking load model, a crowd load model, and an equivalent number of persons calculation method. When calculating the vertical crowd load, the crowd load is considered in two cases according to the crowd density: when the crowd density is 0.5–0.8 p/m^2^, according to Equation (17); and when the crowd density is 1 p/m^2^, according to Equation (18). Both of these are as follows:

(17) $$N_{p}=10.8\sqrt{\xi \cdot n},$$

(18) $$N_{p}=1.85\sqrt{n},$$

where in this load model, *d* is the crowd density; *n* is the number of pedestrians on the bridge and *n* = *d* × *S*, where *S* is the loading area; *P* is the magnitude of the single-person dynamic load; *f*_0_ and *ξ* are the fundamental frequency and damping ratio of the bridge, respectively; and *ψ* is the load reduction factor.

**Figure 3:**
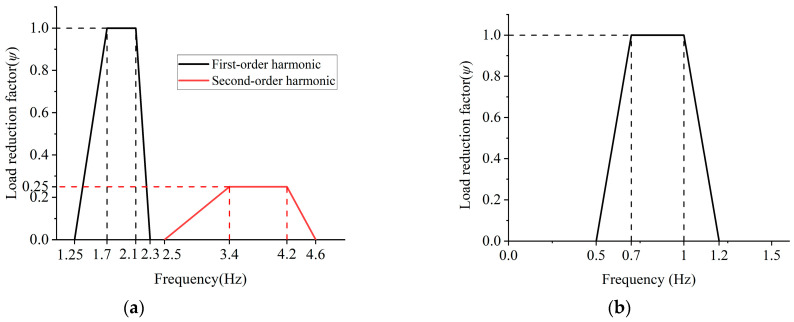
The value regulations for *ψ* in EN 03. (**a**) Vertical and longitudinal directions; (**b**) horizontal direction [39].

The same equation is used for the vertical, longitudinal, and lateral loads in the German guideline EN 03 [39] and China’s Standard for vibration of buildings [40]. After the introduction of the equivalent pedestrian density *n*′ = *N*_p_/*S*, their expressions for crowd loads are identical to those of Sétra. In addition, all three codes take into account the effect of pedestrian density and the difference between the pedestrian step frequency and the bridge self-oscillation frequency by means of a discount factor. The differences are in the value of the reduction factor and in the classification of the traffic levels (in general, a threshold value for the equivalent number of pedestrians is used for the crowd density *d* = 1 p/m^2^). The value regulations for *ψ* and *P* in EN 03 are shown in Figure 3 and Table 4.

Although the deterministic time-domain model is deficient in theoretical rigor due to the neglect of non-periodic and narrow-band stochastic factors, it is still chosen by the national codes since the testing methods for the main parameters of the model have been gradually improved and matured and the model has the significant advantage of simplicity of form. It is not difficult to see that the study of pedestrian loads, in addition to ensuring reasonableness and correctness, should also have simple and easy-to-use characteristics. The focus of crowd loading research is to accurately describe the synergy between individuals in a crowd on the basis of single-person loading, i.e., the extent to which the crowd maintains synchronization of movements. However, this issue is still in the research stage and progress is lagging behind engineering practice, with no recognized model developed. BRO 2004 and ISO 10137 regulations for crowd loading still use results from classical vibration theory. Even the relatively advanced French Sétra is based on more ideal assumptions and fails to reveal the synergies between different individuals and the distribution characteristics of control elements.

## 3. Evaluation of Human-Induced Vibration Comfort on Pedestrian Bridges

To some extent, the comfort evaluation of pedestrian bridges overlaps with the analysis of the anthropogenic vibration response. The process of comfort evaluation is to calculate the maximum dynamic response on the basis of determining the dynamic characteristics of the bridge and correctly modeling the pedestrian loads, and to evaluate this response by adopting reasonable indicators and limit values. Therefore, in addition to the modeling of pedestrian loads, another issue is the determination of response indicators and their limit values.

The research on human comfort caused by vibration started early. Mallock found that when the amplitude is small and the acceleration is large it feels unpleasant to the human body through the human body’s perception of vibration from 10 to 15 Hz. He took 0.1 m/s^2^ as the acceleration that the human body can perceive, and 0.5 m/s^2^ as the limit value of the acceleration that makes people annoyed [41], and these values are still adopted by the design codes of some countries until today. Goldman [41] collated the results of other researchers and classified the human body’s perception of vibration into three categories according to the acceleration: detectable, uncomfortable, and intolerable. The limit of discomfort is 0.46 m/s^2^. Dieckmann [42] investigated the sensitivity of the human body to vertical and horizontal vibrations up to 4 Hz and noted that the human body is more sensitive to horizontal vibrations. Although these studies do not directly address pedestrian bridge vibration, these reports provide a useful reference for comfort issues on pedestrian bridges. Wright and Green [43] measured the vibration amplitude of a large number of highway bridges, and according to Goldman’s grading scale one-quarter of these bridges would have reached the intolerable level. However, these bridges did not have comfort issues. Wright argued that the actual vibration of the bridges was very short and that the comfort limits obtained by using prolonged vibration were not applicable. Accordingly, Lenzen [44] argued that the most important factor affecting comfort is the damping ratio, but this view did not seem to be picked up by later studies. Leonard [45] carried out a laboratory study on the comfort of stationary and walking pedestrians using simply supported beam bridges subjected to simple harmonic vibration and concluded that stationary pedestrians are more sensitive to vibration. Subsequently, they suggested that the stationary vertical acceleration limit should be used as the lateral comfort limit. Later, Smith [46] conducted a similar study and classified three categories of comfort based on the human perception of vibration and gave limit values for each. It is worth noting that the acceleration limits proposed by Smith are much larger than the values proposed by Leonard. Given this, Blanchard et al. [20] took the average value of 0.5$\sqrt{f}$(*f* is the bridge fundamental frequency) of their research results as the acceleration limit value, and this result was later adopted by the design specifications of pedestrian bridges in many countries. Irwin [47] combined the experimental and measured data of others and proposed to use the root mean square acceleration as the comfort evaluation index, which was also applied by the specifications of many countries.

From the above studies, it can be seen that the comfort of pedestrian bridge vibration basically focuses on vertical vibration, and the comfort of lateral vibration is less studied and mainly concentrated on in high-rise buildings. Nakamura [48] studied the lateral comfort of footbridges under the action of crowd loads. He suggested taking 1.35 m/s^2^ as the lateral comfort limit. From an engineering experience perspective, this limit is too large and inappropriate.

When the density of pedestrians on the bridge is high, in addition to mutual constraints among pedestrians themselves, the progressively increasing dynamic response of the bridge will also enhance synchronization among the crowd. This occurs as pedestrians subconsciously adjust their step frequency and phase to match the bridge’s vibrations, ultimately locking into a state of synchronization with the structure. This leads to escalating vibrations and triggers dynamic instability. This phenomenon of excessive vibration generally occurs in transverse vibration, i.e., the transverse dynamic instability lock-in phenomenon. Even in the absence of comfort anomalies, there is still a possibility of lateral locking of the bridge, which involves the limit state of the load-carrying capacity of the bridge structure. The large vibration of the Millennium Bridge in London in 2000 was caused by this reason, and since then many scholars have begun to pay attention to the problem of transverse dynamical instability in pedestrian bridges. Fujino et al. [49] observed artificially induced large-amplitude transverse vibration of real bridges under congested conditions and investigated the effect of pedestrian lateral resonance force and lateral vibration due to walking synchronization. They observed that there is a correlation between lateral displacement and lateral force, while no correlation was observed between the lateral and vertical displacement, and concluded that lateral vibration phenomenon may also be induced by direct resonance phenomenon alone. Ingólfsson et al. [50] proposed a frequency- and amplitude-dependent pedestrian-induced transverse force stochastic load model for the simulation of footbridges, but the model cannot take into account nonlinear modal coupling, the possibility of parametric excitation, and excitation of transverse modes through vertical loading. At present, there are three main theories to explain the lateral dynamic instability problem:

1. Power interaction theory, based on some kind of model between the bridge structural motion and pedestrian walking;
2. Direct resonance theory, which considers that the pedestrian excitation frequency happens to be equal to the frequency of a certain modal state of the bridge, which causes a resonance;
3. Internal resonance theory, which considers that the structural nonlinearities cause different modes to enter into an internal resonance state.

More widely used is the negative damping model proposed by Dallard et al. based on the power interaction theory [51]. The pedestrian footstep force adapted to the vibration during lateral vibration of the structure is in the same direction as the velocity, providing negative damping to the bridge structure, which is canceled out with the inherent (positive) damping of the bridge itself. When the damping force *F_D_* is greater than the pedestrian’s footstep force *F_e_*, the vibration is attenuated; when *F_D_* is equal to *F_e_*, the structural damping disappears, the dynamic response suddenly increases, and the bridge reaches the critical state of lateral lock-in. Dallard et al. used this model and the actual data of the Millennium Bridge to derive the critical number equation. According to the test results of the Millennium Bridge, the pedestrian lateral force is approximately proportional to the dynamic response of the bridge. Dallard et al. further proposed that the pedestrian lateral force is directly proportional to the lateral vibration velocity at the location, which can be expressed by the introduction of the lateral walking force coefficient *k* and the local velocity *V_l_*, *αF_l_* = *kV_l_*, where *k* needs to be obtained by the measurement of actual bridges. From this, the test results of the Millennium Bridge in London are approximately 300 N·s/m. Let the mode function of the transverse vibration mode under consideration be *φ* (*x*), where *φ_i_* is the value of the mode function at point *x_i_* where the *i*th pedestrian is located, and then *φ_i_ = φ (x_i_)*. The relationship between the local velocity *V_l_* and the modal velocity *v* is *V_l_ = φ_i_v*, at which point the contribution of the *ith* pedestrian to the modal force *αφ_i_ F_l_* can be further expressed as *kφ_i_*^2^*v*. According to the negative damping model, the structure maintains its dynamic stability under the condition that *F_D_* − *F_e_* > 0, i.e.,:

(19) $$4\pi fM^{*}\xi -k\sum_{i=1}^{n}\varphi_{i}^{2}>0,$$

where M* and *ξ* are the modal mass and modal damping ratio in this mode, respectively; and *f* is the modal frequency. When *n* pedestrians are distributed on the bridge, the number of pedestrians on the bridge of *dL* length is *dn = (n/L)⋯dL*, which is obtained through the following:

(20) $$\sum_{i=1}^{n}\varphi_{i}^{2}={\int_{0}^{n}\left[\varphi \left(x\right)\right]}^{2}dn=\frac{n}{L}{\int_{0}^{n}\left[\varphi \left(x\right)\right]}^{2}dL=\frac{n}{2},$$

by bringing Equation (20) into Equation (19), the maximum number of pedestrians to maintain the dynamic stability of the structure can be derived as follows:

(21) $$n_{\max}=\frac{8\pi fM^{*}\xi }{k}.$$

In fact, the assumption that the excitation force of the Dallard model grows linearly with the response velocity is not consistent with the facts because some pedestrians slow down or even stop walking when excessive vibration occurs in the bridge, resulting in a reduction in the excitation force. Based on this, Nakamura considered the self-limiting nature of the pedestrian synchronization phenomenon and used a fractional function instead of Dallard’s linear factor *v* to give a similar model closer to reality. In addition, one limitation of Dallard’s model was that it was not clear whether the value of *k* could be generalized to other bridges, a problem that Nakamura [48] did not address—his parameters were also estimated from experiments and were difficult to obtain at the design stage. Yuan [52] considered both synchronized and unsynchronized pedestrian excitations, incorporating synchronized phase difference between crowd loads and structural vibration, and gave a relevant model, but it essentially still relied on empirical parameters. Roberts [53], on the other hand, treated the crowd as a vibrating system, gave the equations of motion for pedestrians and bridges, and solved the equations by coupling the action and reaction forces. However, the assumptions and values of their models lacked rigorous justification, which led to many unreasonable aspects. Dey [54] systematically studied the lateral vibration standards for pedestrian bridges and pointed out that some current design codes (such as ISO 10137 and Sétra) may fail because they do not explicitly consider the pedestrian–structure interaction. At present, the problem of lateral lock-in is an urgent problem in engineering practice, and there is still a large research space. Dallard’s model was adopted because of its simple form, but it is only a stopgap measure.

According to related research, two types of methods are mainly derived for the evaluation and control of human-caused vibration comfort of pedestrian bridges. One is the method of avoiding sensitive frequency, i.e., determining the intrinsic frequency of the bridge so that it tries to avoid the frequency interval of the pedestrian load (sensitive frequency interval) to avoid the modal resonance triggered by the pedestrian load. China’s CJJ69-95 [9] adopts this method. Second is the method of limiting the dynamic response. When the inherent frequency of the footbridge cannot avoid the sensitive frequency interval, the bridge can meet the comfort requirements by controlling the dynamic response of the bridge under the pedestrian load. The human body is most sensitive to the perception of acceleration, so the limiting dynamic response method generally uses acceleration as a comfort evaluation index. Although the possibility of excessive vibration leading to pedestrian synchronization also exists in the vertical direction, the currently observed events occur among the transverse vibrations. Therefore, the transverse acceleration limits are generally stricter than the vertical direction, not only to ensure more sensitive transverse comfort than the vertical direction but also to avoid excessive vibration leading to pedestrian–bridge interaction and lateral lock-in. Most current national codes basically use the limiting dynamic response method (or in combination with the avoidance of sensitive frequencies method) to evaluate or control bridge comfort.

In 1978, the British BS 5400 specification [36] first began to consider the comfort of pedestrian bridges, and the comfort evaluation index used is peak acceleration and the basis of the calculation is the vertical fundamental frequency. According to its provisions, when the vertical fundamental frequency of the bridge *f*_v_ > 5 Hz, no comfort calculation is required; when 1.5 ≤ *f*_v_ ≤ 5 Hz, comfort calculation should be carried out, and its limit value of vertical vibration acceleration is 0.5$\sqrt{f_{v}}$. The BS 5400 specification does not provide the limit value of the transverse dynamic response, and later supplemented it by stipulating that the maximum value of transverse acceleration should be less than 0.25 m/s^2^. In fact, the specification has now been abandoned in the UK and replaced by the Eurocode, but its contents are still used in other countries that have borrowed from it, such as South Africa. The European EN1990 [7] suggested that the pedestrian step frequency is the same as a certain order of the bridge structure’s self-oscillation frequency, which is prone to resonance, and the vibration response needs to be analyzed. Although the pedestrian load model and calculation method are not given, the specification makes specific provisions on the basis and index of comfort calculation; if the bridge vertical fundamental frequency *f*_v_ > 5 Hz and lateral and torsional fundamental frequency *f*_h_ > 2.5 Hz, no comfort calculation is needed, otherwise, calculation is required. The comfort index adopts the peak acceleration, which stipulates that the vertical maximum acceleration shall not exceed 0.7 m/s^2^ and the lateral maximum acceleration shall not exceed 0.2 m/s^2^ in normal use and shall not exceed 0.4 m/s^2^ when full of people. The draft revision of EN 1990 is even more stringent: the smaller of 0.5$\sqrt{f_{v}}$ and 0.7 m/s^2^ of BS 5400 is recommended for vertical peak accelerations and the smaller of 0.4$\sqrt{f_{h}}$ and 0.15 for transverse. The sensitive frequency intervals for the comfort calculation of footbridges specified in Sétra are consistent with EN 1990, and the peak acceleration is also chosen as the comfort evaluation index. It divides the comfort level of footbridges into four levels, which is also chosen by the later EN 03 and listed in Table 5. EN 03 requires for comfort calculations to be carried out when the vertical and longitudinal intrinsic frequency of the structure is 1.25 Hz ≤ *f_i_* ≤ 2.3 Hz or when the transverse frequency is 0.5 Hz ≤ *f_i_* ≤ 1.2 Hz. If vertical or longitudinal second-order walking loads are considered then the corresponding frequency interval is extended to 1.25 Hz ≤ *f_i_* ≤ 4.6 Hz, and no second-order loads are considered in the horizontal direction. However, it is clearly stated in the guidelines that the second-order simple harmonic loads may arise, causing vertical vibrations in the structure, but up to now no vibrations induced by second-order simple harmonic loads on pedestrian bridges have ever been documented. It should be noted, however, that Sétra actually requires that the lateral acceleration range be limited to 0.1 m/s^2^ at all comfort levels, or that the critical number of lateral instabilities be calculated according to Equation (21) to prevent any lateral lock-in, whereas EN 03, in addition to adopting Equation (21), defines the conditions for triggering the locking phenomenon by means of the lateral acceleration amplitude: *a*_lock in_ = 0.1–0.15 m/s^2^. The distribution characteristics of the Chinese code CJJ69-95 only stipulates that the vertical self-vibration frequency of the superstructure of a footbridge should not be less than 3 Hz and does not address lateral vibration and comfort indexes.

**Figure 4:**
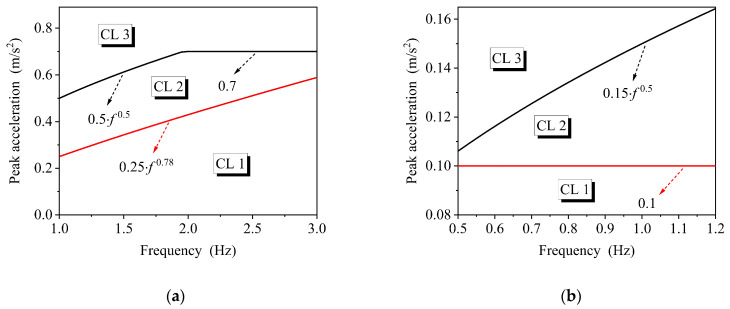
Comfort criteria in Chinese consultative draft of new code. (**a**) Vertical direction; (**b**) horizontal direction [40].

The release of the draft for consultation in 2017 based on CJJ69-95 is seen to be expected to fill the gaps in the design codes for footbridges in China. It suggests that for beam or aluminum-alloy structures the vertical intrinsic frequency ≥ 3 Hz and lateral intrinsic frequency ≥ 1.2 Hz, otherwise, comfort calculation is required; other structural forms are required to calculate the vertical 1.25–3 Hz and lateral 0.5–1.2 Hz model, and the peak acceleration is used as an indicator to classify the three comfort levels (CL3 is unacceptable), as shown in Figure 4.

**Figure 5:**
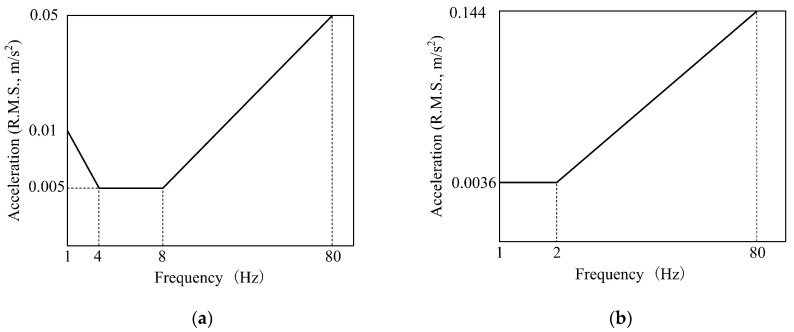
ISO 10137 comfort benchmark curve. (**a**) Vertical direction; (**b**) horizontal direction [38].

The comfort index chosen by BRO 2004 and ISO 10137 is root mean square acceleration(*a*_RMS_). BRO 2004 stipulates that if the vertical fundamental frequency of a footbridge is > 3.5 Hz, no comfort calculation can be carried out; otherwise, it should be calculated, and the vertical *a*_RMS_ should be calculated: *a*_RMS_ ≤ 0.5 m/s^2^. ISO 10137 gives a vibration comfort benchmark curve, as shown in Figure 5. The comfort standard is 60 times this benchmark curve, and below the curve the comfort standard is achieved.

## 4. TMD Design for Controlling Human-Induced Vibration

Frequent and substantial vibrations not only lead to comfort problems but may also lead to fatigue problems in structural materials. The vibration damping of pedestrian bridges generally adopts the additional damping method, i.e., adding damping energy dissipation devices to the main structure to dissipate the vibration energy of the main structure [19,55]. At present, most of the additional damping used in pedestrian bridges are passive energy dissipation devices, such as viscous dampers, tuned mass dampers, tuned pendulum dampers, tuned liquid dampers, etc., and the more commonly used is the tuned mass damper (TMD) [56,57].

**Figure 6:**
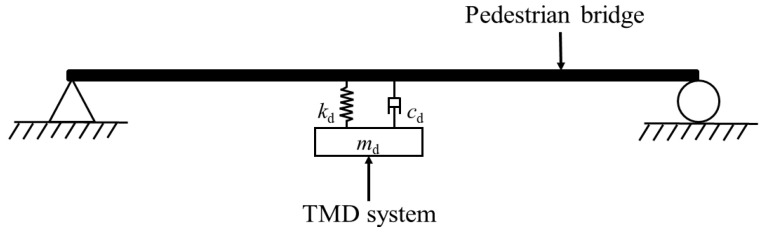
A pedestrian bridge with TMD system [58].

The popular TMD consists of a mass unit, a spring unit, and a damping unit. The mass unit of the TMD is used to provide the inertia force, which interacts with the structure; the spring unit plays the role of frequency tuning and resetting, which is used to tune the frequency of the TMD and the structure so that the TMD can achieve the optimal homodyne condition; and the damping unit plays the role of energy dissipation. When the TMD produces a relative motion concerning the structure, the energy of the external excitation on the structure is transferred to the TMD, and the TMD can dissipate energy through the damping unit, which is used as a means for the TMD to dissipate energy. The TMD dissipates the energy through the damping unit to dissipate the energy, thus effectively reducing the displacement of the structure [58]. The pedestrian bridge with TMD system is shown in Figure 6.

**Figure 7:**
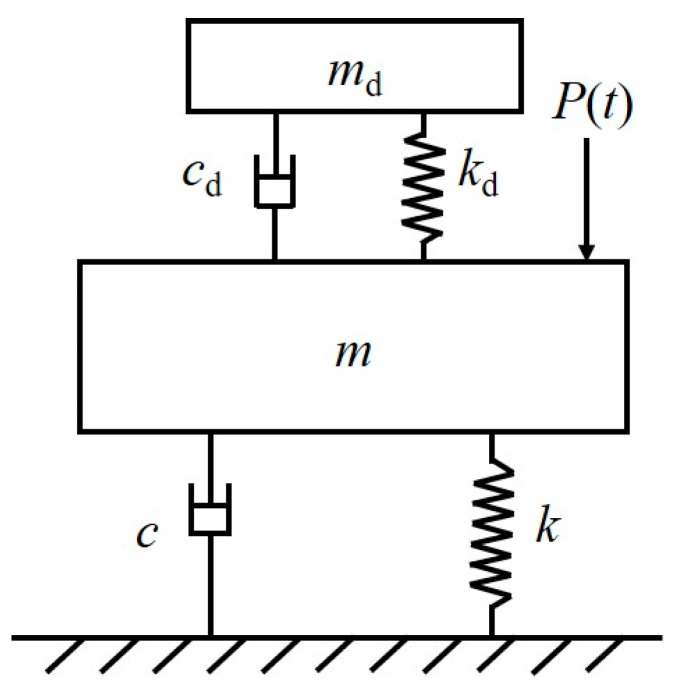
Mechanical model of 2-DOFs system [57].

**Figure 8:**
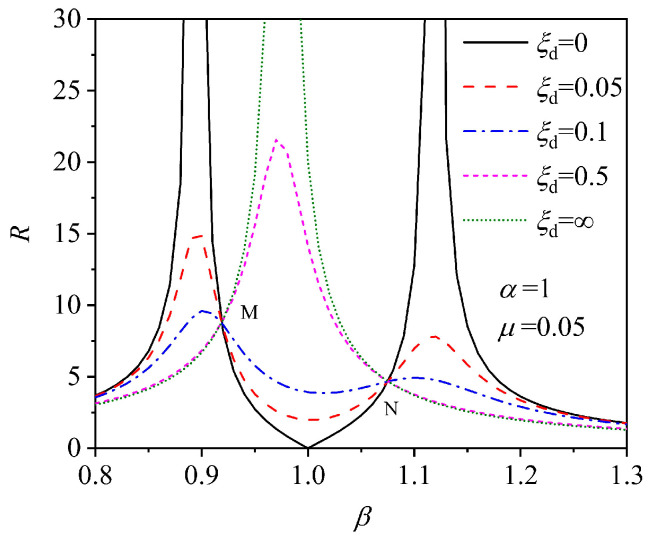
Relationship between amplification factor *R* versus frequency ratio *β* [59].

The human-induced vibration of a pedestrian bridge belongs to the vibration problem of a multi-degree-of-freedom system, but the TMD can only control the vibration for one of the first-order modes. Therefore, when TMD design is carried out a certain order mode under consideration is generally extracted, and the multi-degree-of-freedom problem is transformed into a single-degree-of-freedom problem. Afterwards the TMD is attached to this single-degree-of-freedom system so that it constitutes a double-degree-of-freedom system as shown in Figure 7, and the equations of motion of this system are:

(22) $$\left\{\begin{array}{l} m\ddot{x}\left(t\right)+c\dot{x}\left(t\right)+kx\left(t\right)-c_{d}\left[{\dot{x}}_{d}\left(t\right)-\dot{x}\left(t\right)\right]-k_{d}\left[x_{d}\left(t\right)-x\left(t\right)\right]=P\left(t\right)\\ m_{d}{\ddot{x}}_{d}+c_{d}\left({\dot{x}}_{d}-\dot{x}\right)+k_{d}\left(x_{d}-x\right)=0 \end{array}\right.,$$

where *m*, *c*, and *k* are the mass, damping coefficient, and stiffness of the primary structure, respectively; *m_d_*, *c_d_*, and *k_d_* are the mass, damping coefficient, and stiffness of the TMD, respectively; x, $\dot{x}$ and $\ddot{x}$ are the displacements, velocities, and accelerations of the primary structure, respectively; and $x_{d}$, ${\dot{x}}_{d}$ and ${\ddot{x}}_{d}$ are the displacements, velocities, and accelerations of the TMD. If the damping of the main structure itself is not taken into account, the expression for the power amplification factor *R* with the additional TMD is given by:

(23) $$R=\sqrt{\frac{{\left(\alpha^{2}-\beta^{2}\right)}^{2}+{\left(2\xi_{d}\alpha \beta \right)}^{2}}{{\left[\left(\alpha^{2}-\beta^{2}\right)\left(1-\beta^{2}\right)-\alpha^{2}\beta^{2}\mu \right]}^{2}+{\left(2\xi_{d}\alpha \beta \right)}^{2}{\left(1-\beta -\mu \beta^{2}\right)}^{2}}},$$

where *α* is the frequency ratio between the excitation frequency and the TMD; *β* is the frequency ratio between the excitation frequency and the main structure; *ξ_d_* is the damping ratio of the TMD; and *μ* is the mass ratio between the TMD and the main structure. The optimal design parameters of the TMD (e.g., the mass ratio, the frequency ratio, the damping ratio) are crucial for the damping effect, and the control performance and robustness of the TMD can be effectively improved by optimizing these parameters. According to engineering experience, the mass ratio has a good damping effect at 1% to 5%. Figure 8 shows the correlation curves of amplification factor *R* versus frequency ratio *β* for different damping ratios *ξ_d_* when *α* = 1 and *μ* = 0.05. When *ξ_d_* = 0 and *α* = *β*, the main structure remains stationary, the effects of external load and TMD cancel each other out, and R = 0, then the optimal control effect is achieved. This is the earliest model of a power absorber and the power absorber designed according to this theory has a good damping effect near the resonance frequency of the structure, which theoretically can make the displacement response of the structure zero.

However, since the damping of the TMD is not considered, its control band is very narrow. When the load frequency deviates from the resonant frequency, the vibration amplitude of the main structure will increase substantially, which deteriorates the control effect. Therefore, the damping effect of the main structure has an important relationship with the damping of the TMD. From the figure it can be seen that although the vibration cannot be completely eliminated in the presence of damping of the TMD, and the main structure retains part of the vibration response, the damping frequency band is broadened and the sensitivity to the load frequency is reduced, which makes the robustness of the TMD control improved.

Den Hartog [60] was the first to theoretically investigate the dynamic response of an undampened structure under sinusoidal loading with the addition of a damped TMD, and they pointed out that the introduction of damping improves the problem of deterioration of the control effect when the external load deviates from the resonant frequency. From Figure 8, it can be seen that when *ξ_d_* = 0, *R* is infinite at the two self-resonant frequencies; when *ξ_d_* is infinite, the TMD has no relative displacement concerning the structure, at which time the TMD and the structure actually turn into an undamped system with a mass of (*m* + *m*_d_), and the frequency-domain response of *R* is still infinite at the resonant frequency of this system. Between these two extremes there must exist an optimal damping ratio *ξ*_opt_ for the TMD to minimize the peak of the dynamic response. Notice from Figure 8 that there are two points, M and N, in this series of curves where the power response amplification factor *R* does not change with the damping ratio of the TMD. By adjusting the value of *α* as follows, it is possible to make the M and N vertical coordinates equal, i.e., the optimal frequency ratio *α*_opt_:

(24) $$\alpha_{\mathrm{opt}}=\frac{1}{1+\mu },$$

by bringing Equation (23) into Equation (22) and derivation equal to zero, the formula for the optimum damping ratio can be further derived [61]:

(25) $$\xi_{\mathrm{opt}}=\sqrt{\frac{3\mu }{8{\left(1+\mu \right)}^{3}}}.$$

When the main structural damping is considered, the theoretical formulation of the amplification factor can also be established, but the optimum frequency ratio and damping ratio of the TMD can only be calculated by numerical methods. Ioi and Ikeda [62] used numerical methods to solve the formula for the optimum design parameters of the TMD under sinusoidal loading when structural damping was considered and when the optimization objective was displacement, creating the following formulas:

(26) $$\alpha_{\mathrm{opt}}=\frac{1}{1+\mu }-\left(0.241+1.7\mu -2.6\mu^{2}\right)\xi_{s}-\left(1.0-1.9\mu +\mu^{2}\right)\xi_{s}^{2},$$

(27) $$\xi_{\mathrm{opt}}=\sqrt{\frac{3\mu }{8\left(1+\mu \right)}}+\left(0.13+0.12\mu +0.4\mu^{2}\right)\xi_{s}-\left(0.01+0.9\mu +3\mu^{2}\right)\xi_{s}^{2},$$

where *ξs* is the main structural damping ratio. When the optimization objective is acceleration, the following are obtained:

(28) $$\lambda_{\mathrm{opt}}=\sqrt{\frac{1}{1+\mu }}+\left(0.096+0.88\mu -1.8\mu^{2}\right)\xi_{s}-\left(1.34-2.9\mu +3\mu^{2}\right)\xi_{s}^{2},$$

(29) $$\xi_{\mathrm{opt}}=\sqrt{\frac{3\mu \left(1+0.49\mu -0.2\mu^{2}\right)}{8\left(1+\mu \right)}}+\left(0.13+0.72\mu +0.2\mu^{2}\right)\xi_{s}-\left(0.19+1.6\mu -4\mu^{2}\right)\xi_{s}^{2}.$$

After that, Warburton [63] studied the values of TMD parameters under various random excitations more systematically, and the results show that the optimal frequency ratio and optimal damping ratio of TMD are less affected by the structural damping ratio, but more affected by the mass ratio. In addition, other scholars such as Tsai and Lin [59] also gave TMD optimal parameter equations similar to the above formulas by numerical analysis, and with the development and maturity of computational means the calculation of TMD optimal parameters is also continuously updated. In fact, since the pedestrian loads in the design of pedestrian bridges are generally modeled by Fourier series then the computation of the optimal parameters of TMD using Equations (24)–(29) is still applicable, and these equations are also recommended as the TMD design equations for practical engineering in Sétra.

Although the control band around the superior frequency is broadened by the introduction of the TMD damping, it is still difficult to adapt to the structural vibration caused by random pedestrian loads. Rana and Soong [64] investigated the effect of TMD misalignment on the control effect, and the results showed that the frequency misalignment has a more significant effect on the parameters than the optimal damping ratio offset. Li et al. [65] showed that the effect of a TMD on the control of the vibration of pedestrian bridges is more significant than the optimal damping ratio offset. The study by Li et al. showed that the effect of a TMD in controlling the vibration of a pedestrian bridge is more sensitive to the accurate estimation of the structural dynamic characteristics, and once the structural frequency does not match the frequency of the TMD, i.e., the phenomenon of “detuning” occurs, then the damping effect will be reduced significantly. Therefore, the control of structural vibration by a single TMD depends on the accurate estimation of structural dynamic properties. However, due to practical factors, the structure often differs from the design when it is realized in engineering, resulting in an error between the actual structure and the theoretical calculation. Even if the dynamic properties of the structure could be accurately estimated, the accumulation of damage during the long-term service will result in the degradation of the structural stiffness, causing a decrease in the structural self-oscillation frequency and an increase in damping. In addition, for some pedestrian bridges the controlled modes may not be monolithic, which cannot be addressed by a single TMD. All these factors will cause robustness degradation of the TMD control effect. Lievens et al. [55] proposed a robust design method for TMDs considering structural and loading uncertainties, which significantly improves the performance of TMDs under different uncertainties by using vibration serviceability requirements as design constraints through the numerical optimization techniques. By increasing the mass and damping of the TMD, the robustness to changes in modal parameters can be improved. In addition, independently tuning the TMD parameters can further reduce the mass of the TMD compared to the conventional Asami formulation [66], but requires higher computational cost.

**Figure 9:**
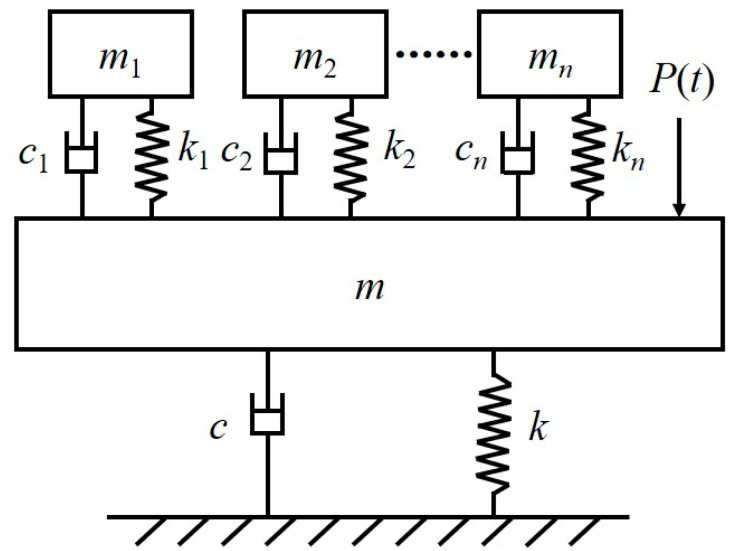
Mechanical model of MTMDs system [67].

To address the limitations of a single TMD, Xu and Igusa [57,67] proposed a multiple tuned mass dampers (MTMDs) system centered on an average frequency and obtained by linearly distributing multiple TMDs with different dynamic characteristics at a certain frequency width. The mechanical model is shown in Figure 9. The MTMDs system is an extension of a single TMD and can simultaneously control the vibration response of multiple modes based on different metrics. The MTMDs system is an extension of a single TMD, which can simultaneously control the vibration response of multi-order modes, and many researchers have analyzed the vibration control problem of MTMDs based on different indices.

Park et al. [68] investigated the optimal dynamic characteristics of the uniformly distributed mass ratio system and the linearly distributed mass ratio system based on the assumptions that the uniformly distributed frequency for MTMDs and the damping ratio of each TMD are equal, respectively. The effect of individual TMD failure on the overall performance of the MTMD is also analyzed. Their results show that the uniformly distributed mass system is more effective in reducing the peak dynamic amplification factor under harmonic loading, while the linearly distributed system is more robust in frequency misalignment and the uniformly distributed system is more reliable when an individual damper fails. Wang et al. [58] compared the effectiveness of TMDs and MTMDs in controlling the vibration of a long-span steel footbridge. The results showed that the TMD system is effective only when the excitation frequency is basically the same as the intrinsic frequency of the structure, and its damping effect will be limited under a wider bandwidth of excitation frequency. In contrast, the MTMD system shows higher damping robustness, and it can stably reduce the structural vibration under all the crowded random excitation and rhythmic excitation. Therefore, the MTMD system may be a more effective choice for the vibration control of an actual pedestrian bridge. The study by Li et al. [65] further showed that the MTMD system can effectively reduce the vibration response caused by the random walking load of a crowd when controlling the vibration of a pedestrian bridge, and the vibration control effect can be significantly improved by optimizing the design parameters of the MTMD. Especially in the case of a wide frequency distribution of the crowd load, the advantage of the MTMD system is more obvious. Yamaguchi et al. [69] and Abe et al. [70] studied the vibration control of a structure under simple harmonic excitation by using the power amplification factor and regression analysis, respectively, assuming that the mass and damping ratio of each TMD in the MTMD system are equal, and gave the mechanism of MTMD vibration control and the regression solution of the parameters. Kareem et al. [71] studied the MTMD control performance of a primary structure subjected to stochastic excitation and discussed the performance of the MTMD for varying mass and frequency distributions. Jangid [72,73] investigated the dynamic performance of the MTMD system with constant stiffness and damping per TMD by using the mean square displacement ratio as an objective function for the structure with and without the MTMD system set up and discussed the effectiveness of the control of the vibration problem of a side-torsion coupled structure subjected to white noise excitation, in this case based on the criterion of minimization of the mean square displacement ratio. Thereafter, Jangid [74] gave an empirical design formulation for the optimal parameters of the MTMD of an undamped structure under base acceleration excitation with the help of a regression analysis technique.

Li [75] systematically studied and summarized the MTMD. He proposed five MTMD models based on the possible combinations of stiffness, mass, damping, and damping ratio of MTMDs, and comprehensively evaluated the control performance of these five modeled MTMDs based on the displacement and acceleration amplification factors. Assuming that the intrinsic frequencies of the *n* TMDs are linearly and uniformly distributed around the frequencies of the controlled modes of the structure *ω*_0_, and *ω*_t_ is the average frequency of the MTMD system, then the frequency increment of the *jth* TMD is:

(30) $$\Delta \omega =\omega_{a}\left(j-\frac{n+1}{2}\right)\frac{\beta }{n-1}.$$

The frequency of the *j*th TMD can be obtained using *ω_j_* = *ω*_t_ + Δ*ω*, where *β* = (*ω*_t_ − *ω*_1_)/*ω*_t_ is the bandwidth. After introducing the center frequency ratio *f* = *ω*_t_/*ω*_0_ of the MTMD system, the frequency ratio *r_j_* of the *j*th TMD to the structurally controlled mode can be expressed as:

(31) $$r_{j}=f\left[1+\left(j-\frac{n+1}{2}\right)\frac{\beta }{n-1}\right].$$

If the average damping ratio of the MTMD system *ξ*_t_ and its modal mass ratio to the primary structure *μ* are, respectively:

(32) $$\mu =\frac{\sum_{j=1}^{n}m_{j}}{m_{0}},$$

(33) $$\xi_{a}=\frac{\sum_{j=1}^{n}\xi_{j}}{n},$$

then the mass ratio *μ_j_* and damping ratio *ξ_j_* can be calculated for the *jth* TMD. The five models and the corresponding computational results are shown in Table 6. The results of the numerical analysis show that the effectiveness and robustness of the MTMD-1 and MTMD-4 controls are better. In engineering practice, the MTMD-1 model is more widely used due to its simple fabrication. Li’s research results greatly promoted the application of MTMD systems in engineering structures, but they have shortcomings. For a given mass ratio *μ*, the optimal average damping ratio of the MTMD decreases with the number of TMDs *n*, and after *n* reaches a certain value the optimal average damping ratio will tend to zero. At this point, the MTMD system will lose its significance, which will constrain the value of *n* or *μ* in the design of larger volume structures. To address this issue, Li and Liu [76] developed the research results by using a uniform distribution of system parameters (abandoning the assumption of a uniform frequency distribution) to give a variety of optimal mean damping ratio models that do not converge to zero, which expands the optionality of MTMD design in engineering practice.

## 5. Conclusions and Outlooks

The core issue of pedestrian-induced vibrations in footbridges is addressed in this paper through a systematic review of major research advances. In terms of pedestrian load models, the modeling methods for single-person loads and crowd loads are described in detail: single-person loads include deterministic time-domain models, stochastic models, and frequency-domain models. Among them, deterministic time-domain models are widely adopted in design codes of various countries due to their mature parameter determination and simple form, while stochastic and frequency-domain models, although closer to reality, remain primarily in the theoretical research stage due to theoretical complexity. Crowd loads are classified into different types according to pedestrian density and states (such as group walking, low-density free walking, high-density flow, and lateral lock-in), with the modeling focusing on describing inter-individual coordination, and relevant models are still in the exploratory stage.

In terms of comfort evaluation, acceleration is defined as the core indicator directly related to pedestrian comfort, and the design codes of various countries generally adopt a combined control method of avoiding sensitive frequency intervals and limiting dynamic responses. Vertical vibrations can be ensured to meet comfort requirements by limiting the acceleration amplitude; lateral vibrations require stricter limits due to the possible lateral lock-in causing dynamic instability, and the critical number of people for instability needs to be calculated. In this study, the limit standards for vertical and lateral accelerations in codes of different countries were compared, presenting the current practical status of comfort evaluation.

Furthermore, we review the vibration reduction design theory of TMDs, including the composition, action principle, and optimal parameter calculation method of TMDs, and mention the advantages of MTMDs in addressing multi-mode vibrations. These vibration reduction technologies provide effective means to solve the problem of excessive vibrations in footbridges.

From the perspective of the field’s current status, research on pedestrian-induced vibrations in footbridges has made significant progress in the construction of practical models (such as deterministic load models) and engineering vibration reduction measures (such as TMD application), with some achievements incorporated into design codes to guide practice. However, existing research still has limitations: the mechanism of inter-individual coordination in crowd loads is not fully clarified and models involve many simplifications; the calculation of the critical number of people for lateral instability depends on the measured parameters of specific bridges, lacking universality; TMDs are relatively sensitive to frequency mismatch, and their robustness needs to be improved when dealing with random pedestrian loads.

The significance of this paper lies in systematically integrating the research achievements of pedestrian load modeling, comfort evaluation, and vibration reduction design, clearly presenting the research framework and status of this field. It not only provides referable theories and methods for engineering designers but also points out the direction for follow-up research—such as deepening the refined modeling of crowd loads, improving the control standards for lateral vibrations, and optimizing vibration reduction technologies to adapt to complex loads. Future research needs to further balance theoretical rigor and engineering practicality, promoting the continuous improvement of footbridge design in terms of safety and comfort.

## Figures and Tables

**Table 1:** DLF values proposed by scholars.

Reference	Direction	DLF	Step Frequency
Blanchard et al. [20]	vertical	*α*_1_ = 0.257	<4 Hz
Bachmann et al. [21]	vertical	*α*_1_ = 0.37, *α*_2_ = 0.1, *α*_3_ = 0.12, *α*_4_ = 0.04, *α*_5_ = 0.08	2 Hz
	longitudinal	*α*_1_ = 0.039, *α*_2_ = 0.0204, *α*_3_ = 0.026, *α*_4_ = 0.083, *α*_5_ = 0.024	2 Hz
	lateral	*α*_1_ = 0.039, *α*_2_ = 0.01, *α*_3_ = 0.043, *α*_4_ = 0.012, *α*_5_ = 0.015	2 Hz
	vertical	*α*_1_*=* 0.4–0.5, α_2_ = α_3_ = 0.1	2.0–2.4 Hz
Allen et al. [23]	vertical	*α*_1_ = 0.5, *α*_2_ = 0.2, *α*_3_ = 0.1, *α*_4_ = 0.05	1.6–2.4 Hz
Petersen [25]	vertical longitudinal lateral	*α*_1_ = 0.518, *α*_2_ = 0.058, *α*_3_ = 0.041 *α*_1_ = 0.408, *α*_2_ = 0.079, *α*_3_ = 0.018 *α*_1_ = 0.073, *α*_2_ = 0.138, *α*_3_ = 0.018	2.5 Hz 2 Hz 1.5 Hz
Kerr et al. [18]	vertical	*α*_1_ = −0.2649 *f_p_*^3^ + 1.3206 *f_p_*^2^ − 1.7597 *f_p_* + 0.7613, *α*_2_ = 0.07, *α*_3_ = 0.06	-
Young [24]	vertical	*α*_1_ = 0.41(*f_p_* − 0.95) ≤ 0.56, *α*_2_ = 0.069 + 0.0056 *f_p_*, *α*_3_ = 0.033 + 0.0064 *f_p_*, *α*_4_ = 0.013 + 0.0065 *f_p_*	1.0–2.8 Hz
Chen et al. [26]	vertical	α_v1_ = 0.2817 *f_p_* − 0.00792, α_v2_ = 0.1675, α_v3_ = 0.1085, α_v4_ = 0.0988, α_v5_ = 0.0783	1.5–2.5 Hz

**Table 2:** First five-order DLFs of main harmonics and subharmonics [29].

Order	Main Harmonic		Subharmonic
	Mean	Standard Deviation	
1	*μ*_1_	0.16 *μ*_1_	0.026 *α*_1_ + 0.0031
2	0.07	0.03	0.074 *α*_1_ + 0.01
3	0.05	0.02	0.012 *α*_1_ + 0.016
4	0.05	0.02	0.013 *α*_1_ + 0.0093
5	0.03	0.015	0.015 *α*_1_ + 0.0072

**Table 3:** Pedestrian load models in some design codes.

Code	Load Model
BS 5400 [36]	$\mathrm{Single}:\;F_{p}\left(t\right)=G\cdot \alpha \sin\left(2\pi ft\right)$
BRO 2004 [37]	$\mathrm{Crowd}:\;F_{p}\left(t\right)=150\sqrt{0.1BL}\sin\left(2v\right)$
ISO 10137 [38]	$\mathrm{Single}:\;F_{p}\left(t\right)=G\left[1+\sum_{i=1}^{k}\alpha_{i}\sin\left(2\pi if_{p}t+\phi_{i}\right)\right]$ $\mathrm{Crowd}:\;F\left(t\right)=n\cdot F_{p}\left(t\right)\cdot C\left(n\right)$
Sétra [8]	$\mathrm{Single}:\;F_{p}\left(t\right)=G_{0}+G_{1}\sin\left(2\pi f_{p}t\right)+\sum_{i=2}^{n}G_{i}\sin\left(2\pi if_{p}t-\varphi_{i}\right)$ $\mathrm{Crowd}:\;F_{p}\left(t\right)=d\cdot P\cdot \cos\left(2\pi f_{p}t\right)\cdot N_{p}\cdot \psi$
EN 03 [39]	$\mathrm{Crowd}:\;F_{p}\left(t\right)=P\cdot \cos\left(2\pi f_{p}t\right)\cdot n^{\prime }\cdot \psi$
GB/T 51228-2017 [40]	$\mathrm{Crowd}:\;F_{p}\left(t\right)=P\cdot \cos\left(2\pi f_{p}t\right)\cdot n^{\prime }\cdot \psi$

**Table 4:** The value regulations for *P* in EN 03 [39].

Direction	*P(*N*)*
Vertical Direction	280
Longitudinal Direction	140
Horizontal Direction	35

**Table 5:** Comfort level defined in Sétra and EN 03 [8,39].

Comfort Level	Degree of Comfort	Vertical (m/s^2^)	Lateral (m/s^2^)
CL 1	Maximum	≤0.5	≤0.15
CL 2	Medium	0.5–1.0	0.15–0.3
CL 3	Minimum	1.0–2.5	0.3–0.8
CL 4	Unacceptable	>2.5	>0.8

**Table 6:** MTMD models proposed by Li [75].

Model	Hypothesis	*μ_j_*	*ξ_j_*
MTMD-1	*k*_1_ = *k*_2_ = … = *k_n_* = *k*_t_, *c*_1_ = *c*_2_ = … = *c_n_* = *c*_t_, *m*_1_ ≠ *m*_2_ ≠ … ≠ *m_n_*, *ξ*_1_ ≠ *ξ*_2_ ≠ … ≠ *ξ_n_*	$\frac{\mu }{r_{j}^{2}\sum_{j=1}^{n}\left(1/r_{j}^{2}\right)}$	$r_{j}\xi_{t}$
MTMD-2	*m*_1_ = *m*_2_ = … = *m_n_* = *m*_t_, *ξ*_1_ = *ξ*_2_ = … = *ξ_n_* = *ξ_t_*, *k*_1_ ≠ *k*_2_≠ … ≠ *k_n_*, *c*_1_ ≠ *c*_2_ ≠ … ≠ *c_n_*	μ/n	$\xi_{t}$
MTMD-3	*m*_1_ = *m*_2_ = … = *m_n_* = *m*_t_, *c*_1_ = *c*_2_ = … = *c_n_* = *c*_t_, *k*_1_ ≠ *k*_2_ ≠ … ≠ *k_n_*, *ξ*_1_ ≠ *ξ*_2_ ≠ … ≠ *ξ_n_*	μ/n	$\frac{n\xi_{t}}{r_{j}\sum_{j=1}^{n}\left(1/r_{j}\right)}$
MTMD-4	*k*_1_ = *k*_2_ = … = *k_n_* = *k*_t_, *ξ*_1_ = *ξ*_2_ = … = *ξ_n_* = *ξ_t_*, *m*_1_ ≠ *m*_2_ ≠ … ≠ *m_n_*, *c*_1_ ≠ *c*_2_ ≠ … ≠ *c_n_*	$\frac{\mu }{r_{j}^{2}\sum_{j=1}^{n}\left(1/r_{j}^{2}\right)}$	$\xi_{t}$
MTMD-5	*c*_1_ = *c*_2_ = … = *c_n_* = *c*_t_, *ξ*_1_ = *ξ*_2_ = … = *ξ_n_* = *ξ_t_*, *k*_1_ ≠ *k*_2_ ≠ … ≠ *k_n_*, *m*_1_ ≠ *m*_2_ ≠ … ≠ *m_n_*	$\frac{\mu }{r_{j}\sum_{j=1}^{n}\left(1/r_{j}\right)}$	$\xi_{t}$

## Data Availability

No new data were created or analyzed in this study.
